# Endoscopic ultrasound-guided access using a lumen-apposing metal stent for the management of duodenal bleeding in a Roux-en-Y anatomy after gastric bypass

**DOI:** 10.1055/a-2239-8326

**Published:** 2024-02-02

**Authors:** Anna Fichtl, Leonard Elad, Marko Kornmann, Thomas Seufferlein, Martin Müller, Martin Wagner, Benjamin M. Walter

**Affiliations:** 1 27197 Department of Internal Medicine I, Ulm University Hospital, Ulm, Germany; 2 27197 Endoscopy Research Unit, Ulm University Hospital, Ulm, Germany; 3 27197 Bariatric surgery, Department of Visceral Surgery, Ulm University Hospital, Ulm, Germany; 4 27197 Department of Visceral Surgery, Ulm University Hospital, Ulm, Germany

A 54-year-old woman with obesity presented to our hospital with symptoms of severe gastrointestinal bleeding. She had undergone bariatric robotic Roux-en-Y gastric bypass 4 months previously. Endoscopic examination revealed no signs of active bleeding in the gastric remnant, but signs of upper gastrointestinal bleeding were evident through the afferent jejunal loop, leading us to suspect that the bleeding originated from the bypassed stomach. After interdisciplinary discussion we decided on an endoscopic ultrasound (EUS)-guided approach to re-enter the bypassed stomach ( Video 1 ).

Endoscopic ultrasound-guided access using a lumen-apposing metal stent for the management of duodenal bleeding in a Roux-en-Y anatomy after gastric bypass.Video 1

Placement of a lumen-apposing metal stent (LAMS) was performed under EUS guidance. The access route to the bypassed stomach was dilated using a through-the-scope balloon to facilitate endoscopic passage 1 . During the endoscopic evaluation of the bypassed stomach, minor gastric ulcerations were observed in the antrum. Before progressing into the bypassed duodenum, the correct position of the LAMS was checked, as there is a risk of intraprocedural LAMS migration 2 . When passing the pylorus into the duodenum, there were signs of active bleeding (Forrest IB) in the descending part of the duodenum, which was successfully treated with two metal clips.

Endoscopic evaluation was performed 2 weeks later after no further signs of recurrent bleeding. Hence, the LAMS was removed with a rat-toothed grasping forceps. The remaining mucosal defect was closed with an over-the-scope clip to reinstate the gastric bypass.

It has been previously demonstrated that endoscopic gastrogastrostomies for endoscopic ultrasound-directed transgastric endoscopic retrograde cholangiopancreatography in patients with Roux-en-Y anatomy are effective and safe 3 . Therefore, we are confident that LAMS can also be effectively utilized for other intra- or transgastric interventions in Roux-en-Y cases, as seen in our unique case of duodenal bleeding.

Endoscopy_UCTN_Code_TTT_1AS_2AG

## Notes

Endoscopy E-Videos https://eref.thieme.de/e-videos
*E-Videos* is an open access online section of the journal *Endoscopy* , reporting on interesting cases and new techniques in gastroenterological endoscopy. All papers include a high-quality video and are published with a Creative Commons CC-BY license. Endoscopy E-Videos qualify for HINARI discounts and waivers and eligibility is automatically checked during the submission process. We grant 100% waivers to articles whose corresponding authors are based in Group A countries and 50% waivers to those who are based in Group B countries as classified by Research4Life (see: https://www.research4life.org/access/eligibility/ ). This section has its own submission website at https://mc.manuscriptcentral.com/e-videos .
